# Maximizing single-pass conversion does not result in practical readiness for CO_2_ reduction electrolyzers

**DOI:** 10.1038/s41467-023-41348-w

**Published:** 2023-09-07

**Authors:** Shashwati C. da Cunha, Joaquin Resasco

**Affiliations:** McKetta Department of Chemical Engineering, The University of Texas at Austin, Austin, TX 78712 USA

**Keywords:** Chemical engineering, Electrocatalysis

## Abstract

The authors comment that maximizing product concentration is a more meaningful target for CO2 electrolyzers than maximizing single-pass conversion.

## Teaser

For many chemical processes, high single-pass conversion of reactants into products reduces the need to separate products downstream. However, low-temperature carbon dioxide electrolyzers that maximize single-pass conversion suffer from low product concentration. Maximizing product concentration is therefore a more meaningful target for CO_2_ electrolyzers than maximizing single-pass conversion.

To integrate electrochemical carbon dioxide reduction (CO_2_R) into the chemicals industry at scale, the outlet streams from CO_2_ electrolyzers must be product rich. Single-pass conversion is becoming increasingly common as a performance benchmark for CO_2_ electrolyzers because it suggests concentrated products and reduced separation energy for many catalytic processes. However, our analysis shows that CO_2_R reactor configurations that maximize single-pass conversion currently suffer from low product concentration in the outlet stream. This is because they restrict CO_2_ flow or operate in acidic cathode environments, which promote considerable hydrogen evolution as a side reaction. For any gas products besides syngas, high single-pass conversion does not signify that separation energy losses have been eliminated, or that product streams are directly suitable as feedstocks for downstream processes. We therefore recommend that researchers targeting CO_2_R scaleup report product concentrations rather than relying on single-pass conversion as an indicator of overall performance. To commercialize CO_2_ electrolysis, maximizing product concentration is a more relevant goal than maximizing single-pass conversion.

## Outlet streams from CO_2_ electrolyzers need gas separations

Electrochemical CO_2_ reduction is a potential low-carbon pathway for producing chemical feedstocks and fuels from renewable electricity, water, and CO_2_^1,2^. CO_2_ electrolyzers can produce value-added chemicals whose industrial demand is in the hundreds of megatons annually^3,4^, including carbon monoxide^5^, formic acid^6^, and ethylene^7^. CO can in turn be electrochemically reduced in a two-stage cascade^8,9^.

State-of-the-art CO_2_ electrolyzers have limited reactant utilization because of the incomplete reaction of CO_2_, as well as CO_2_ crossover due to carbonate acid-base equilibrium. Selectivity is also limited by competition from water reduction via the hydrogen evolution reaction (HER), in which CO_2_ is uninvolved. Gas separations and recycle streams are needed to recover and convert unreacted CO_2_, and to purify products.

**Fig. 1:**
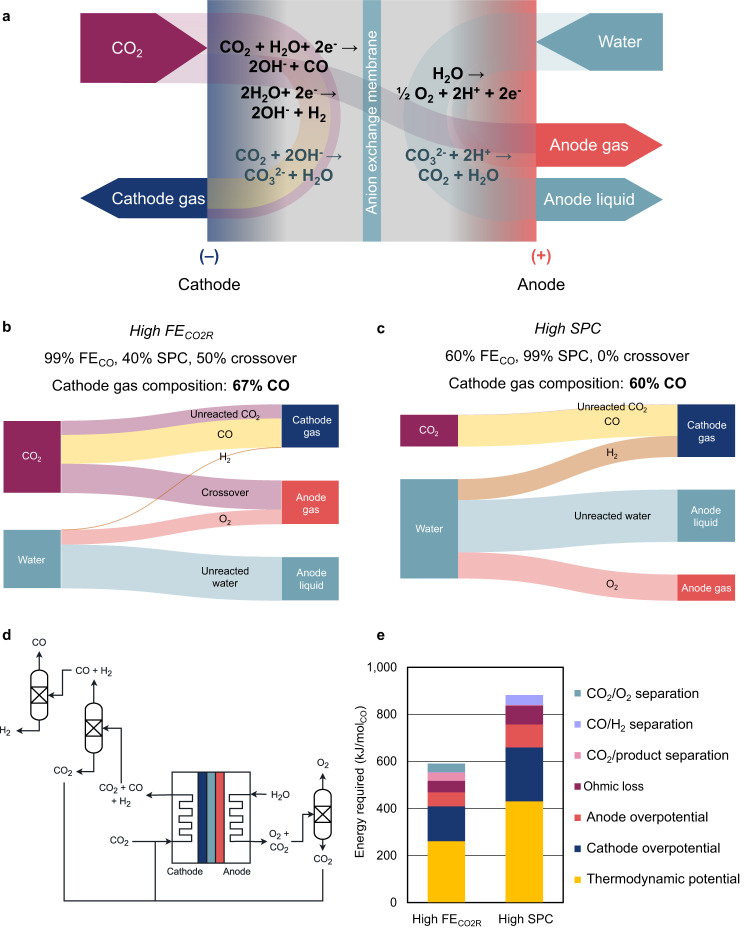
Typical CO_2_ electrolyzer outlet gases. **a** Reaction pathways for a typical CO_2_ electrolyzer reducing CO_2_ to CO in a neutral electrolyte with an anion exchange membrane. Black text indicates heterogeneous redox reactions, while blue indicates homogeneous reactions. **b**, **c** Molar flow rates in a single pass of CO_2_ reduction to CO for example scenarios with (**b**) high Faradaic efficiency towards CO_2_R with realistic parameters and (**c**) high single-pass conversion with optimistic parameters. The total current is the same in both cases. Despite optimistically high single-pass conversion in (**c**), CO concentration in the cathode outlet is decreased. **d** Sample gas separation scheme required for a CO_2_ electrolyzer. **e** Energy required per mole of product for reaction (modeled as overpotential) versus separation (modeled thermodynamically) shows that the high-SPC scenario wastes reactor energy on hydrogen evolution. Process parameters correspond to (**b**) and (**c**).

Typical CO_2_ electrolyzers produce two gas-phase outlet streams, one each at the cathode and anode, which can both contain residual CO_2_ (Fig. 1a). The cathode gas contains gas-phase CO_2_R products, unreacted CO_2_, and hydrogen as a byproduct from the HER. CO_2_ electrolyzers can produce carbon monoxide with 100% molar selectivity^10,11^, so a hydrogen-free tail gas can be produced under optimal conditions. For multicarbon products, state-of-the-art electrolyzers have a molar selectivity of 50% to C_2+_ products, with the co-evolution of 20% H_2_ and 30% C_1_ products at the cathode^12,13^. The scope of this discussion is limited to gas-phase separations, which are necessary for the production of CO or of ethylene, the dominant C_2+_ product. For liquid products like formic acid, ethanol, and acetate, both gas and liquid product streams are formed, which requires an independent analysis of separations.

At the anode, oxygen is produced from water oxidation via the oxygen evolution reaction (OER). The anode outlet also contains CO_2_ that crosses over the membrane via the homogeneous reactions shown in Fig. 1a^14,15^. This crossover arises from acid-base equilibria—hydroxide generated by the cathodic reaction converts dissolved CO_2_ into (bi)carbonate ions in neutral electrolytes, like KHCO_3_ or Cs_2_CO_3_, or alkaline electrolytes such as KOH. (Bi)carbonate anions migrate towards the anode, where they buffer protons generated by OER, regenerating CO_2_ gas. This buffering reaction limits single-pass conversion at most pH^16^. In neutral electrolytes, carbonate ion crossover stoichiometrically consumes 0.5 mol CO_2_/mol e^−^, which results in an anode tail gas consisting of 67 mol% CO_2_ + 33 mol% O_2_. Therefore, both the cathode and anode outlet gases can contain unreacted CO_2_ that must be captured and recycled to the cathode (Fig. 1b, c).

Recycling unreacted CO_2_ requires a gas separation that could be a capital- and energy-intensive process. An electrolyzer with incomplete conversion, imperfect selectivity, and reactant crossover needs at least three pairwise separations: one to separate the target product from byproducts, and two to recycle CO_2_ from the cathode and anode outlets (Fig. 1d). Separation units are typically modeled as pressure-swing adsorption using electrical utilities^3^, but cryogenic distillation or amine scrubbing could be preferable depending on process scale, stream compositions, and available utilities^17^. If extensive downstream separations are required to purify products and recycle CO_2_, the overall energy efficiency of CO_2_R has been argued to suffer unacceptably^15^. To account for separation demands, studies on CO_2_ electrolyzers increasingly report single-pass conversion (SPC):

$${{{{{{\rm{SPC}}}}}}}=\frac{{{{{{\rm{C}}}}}}{{{{{{\rm{O}}}}}}}_{2}\;{{{{{{\rm{moles}}}}}}\; {{{{{\rm{converted}}}}}}\; {{{{{\rm{to}}}}}}\; {{{{{\rm{products}}}}}}}}{{{{{{\rm{C}}}}}}{{{{{{\rm{O}}}}}}}_{2}\;{{{{{{\rm{moles}}}}}}\; {{{{{\rm{fed}}}}}}\; {{{{{\rm{to}}}}}}\; {{{{{\rm{reactor}}}}}}}}$$

In other catalytic processes, high SPC generally suggests improved reactant utilization and hence lower energy to recycle the unreacted feed. Also, downstream applications, like ethylene polymerization, typically require high-purity feedstocks. Since high SPC implies that the products are not diluted in leftover reactant, it is associated with marketable product streams.

## High SPC does not eliminate cathode gas separations

**Fig. 2:**
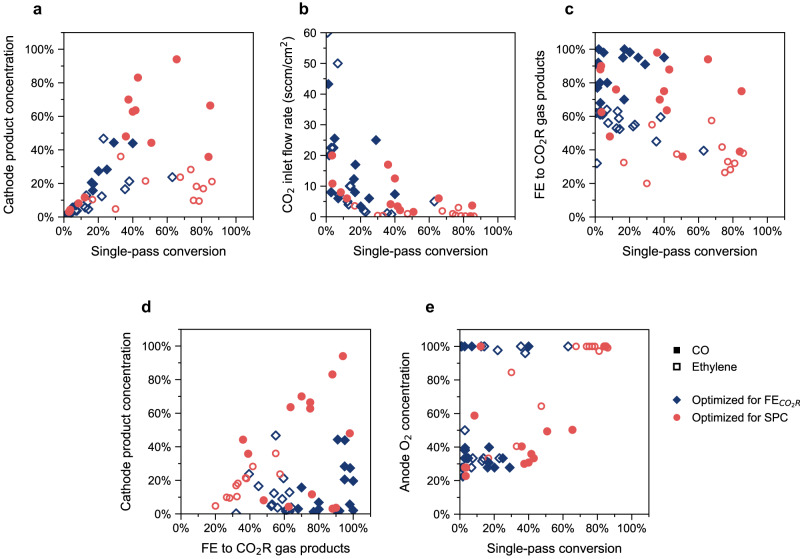
Correlations between single-pass conversion and CO_2_R performance in literature. **a** High single-pass conversion does not result in highly concentrated products that are suitable for downstream applications. **b** High single-pass conversion has been achieved at low feed flow rates, at which hydrogen evolution is the dominant reaction. **c** Faradaic efficiency to CO_2_R trades off with single-pass conversion across various reaction conditions. **d** Concentrated cathode streams are produced by maximizing Faradaic efficiency, which is extremely challenging at high single-pass conversions. **e** Anode gas separations can be minimized across a range of single-pass conversion. Hollow symbols () correspond to ethylene production and filled symbols () represent CO generation. Red circles () correspond to the highest single-pass conversion in a report, and blue diamonds () correspond to the highest FE_CO2R_ in a report; their operating conditions are usually different since selectivity and SPC trade off in current electrolyzer configurations^10,16,18–20,22,27,29–49^.

Molar stream composition is a critical consideration for downstream applications. For instance, thermal reactors are sensitive to reactant partial pressures, so CO_2_R products must often be concentrated for downstream processing. To assess the relationship between SPC and reactor outlet composition, we analyzed data from literature reports targeting state-of-the-art CO_2_ electrolyzer performance (for details, see Supplementary Information, Section 3). These relationships are demonstrated in Fig. 2, with additional representations in Fig. S2 and pH dependence in Fig. S3.

As shown in Fig. 2a, we find that experimental conditions that maximize single-pass conversion do not correspond to concentrated product streams suitable for further reaction. To maximize SPC, studies are often conducted at low inlet CO_2_ flow rates (Fig. 2b). Under these conditions, the partial pressure of CO_2_ drops steeply across the reactor as it is consumed by reaction, resulting in a loss in CO_2_R selectivity in favor of HER^18–20^. In some cases, SPC has also been increased using an acidic electrolyte (e.g. H_2_SO_4_ + K_2_SO_4_), or reverse-biased bipolar membrane to eliminate carbonate formation and crossover. Unfortunately, the high availability of protons in such configurations steers selectivity towards HER over CO_2_R, which is not captured in SPC since CO_2_ plays no direct role in the HER reaction. This tradeoff between Faradaic efficiency (FE_CO2R_) and SPC has been demonstrated for various electrolyzers previously^21–23^, and is reflected across the dataset we analyzed (Fig. 2c). Hence strategies that reduce the need to recycle CO_2_ still require separations to remove H_2_ and concentrate the product for downstream processes. Additionally, electrolyzer energy is wasted on making H_2_ in these scenarios. A comparison of the energy required for reaction and separation (Fig. 1e, Fig. S1) and a recent in-depth analysis^24^ suggest that reactor energy requirements significantly exceed the energetic costs of separations. Hence the energy demand for CO_2_R is dramatically increased at high SPC/ low FE_CO2R_ conditions, even if gas separations are reduced (Fig. 1e). Therefore, high SPC neither eliminates the need for cathode gas separations, nor indicates that products are formed at an improved energy efficiency.

Neither SPC nor FE_CO2R_ directly reflect outlet composition, as indicated by the lack of a clear trend in Fig. 2a, d. While SPC fails to account for HER, FE_CO2R_ and partial current density do not reflect molar flow rates. For C_2+_ products, this problem is especially acute since multiple electron pairs are transferred for CO_2_R, compared to a single pair to make H_2_. For example, a 90% FE to CO with 10% to H_2_ at 100% SPC translates to a stream composition of 90 mol% CO + 10 mol% H_2_. In contrast, 90% FE to ethylene with 10% to H_2_ results in 60 mol% ethylene + 40 mol% H_2_ because of the 12 e^-^ transferred to produce ethylene.

Although high FE_CO2R_ does not perfectly scale to high molar flows, Fig. 2d shows that the most concentrated cathode product is obtained at high FE_CO2R_, which is extremely challenging to achieve at the low flow rates that give high SPC. At high current densities and FE_CO2R_, high crossover and selective reaction manifest in very concentrated cathode products. On the other extreme, crossover can be minimized to produce 100% O_2_ at the anode. However, a pure O_2_ anode stream has been reported across a range of SPC (Fig. 2e), so high SPC is not a necessary condition for low crossover. If minimizing HER is important for CO_2_R scaleup, then SPC to CO_2_R products is a misleading metric that does not truly reflect that a reactor design is practical.

## Electrolyzers for scaleup should report outlet compositions

To pursue industrial relevance for selective CO_2_R, we recommend a careful selection of metrics and operating conditions for measuring and reporting electrolyzer performance. Our analysis shows that SPC does not accurately reflect downstream compositions at the cathode. A realistic representation of separation demand can be provided by mole fractions and outlet flow rates. The concentration of products in the cathode and anode streams indicates the readiness of electrolyzers for downstream applications, informing separation energy and process feasibility. Therefore, we recommend that researchers report cathode and anode product stream compositions. Section 3 of the Supplementary Information outlines the conversions between more common metrics and stream compositions, which can also be used on existing datasets to analyze the effects of experimental variables on product streams.

We suggest that researchers characterize the composition of the anode gas, as has been recommended by Seger and coworkers^25,26^. At present, very few reports on state-of-the-art CO_2_ electrolyzers explicitly quantify the anode gas stream. The combination of complete anode and cathode gas quantification allows the carbon mass balance to be used as an additional experimental validation step. The carbon balance is especially important in alkaline electrolytes that absorb CO_2_, where anode gas quantification can be nuanced. CO_2_R reports in alkaline conditions rarely assess the extent of carbonate formation^27^ or CO_2_ regeneration at the anode, and often report a lower crossover than is physically reasonable. This may be due to the anolyte not being purged of CO_2_, thus excluding carbonate formation from the carbon balance.

We recommend that researchers supply CO_2_ flow rates that correspond to at least the amount of CO_2_ consumed stoichiometrically by CO_2_R at the applied current. Many reports feed a lower CO_2_ molar flow than the chronopotentiometry current. This artificially inflates SPC at the cost of selectivity, since the CO_2_R partial current density is severely reactant limited and HER must compensate for the remaining current. The complete reaction of 1 sccm of CO_2_ accounts for 143 mA of current through a 2-e^−^ pathway making C_1_ products, or 430 mA of current through a 12-e^−^ C_2_ pathway. Therefore, on a 5 cm^2^ cathode where 1 sccm CO_2_ is fed, it is impossible to produce more than 29 mA/cm^2^ of C_1_ products, even in the absence of crossover. In a neutral electrolyte where carbonate is the dominant charge carrier, CO_2_R current is further constrained by the crossover of 0.5 mol CO_2_/mol e^−^. Under these conditions, the partial current density to 12-e^−^ C_2+_ products from 1 sccm CO_2_ on a 5 cm^2^ electrode is at most 22 mA/cm^2^. Most experiments in membrane electrode assemblies (MEAs) are operated at >50 mA/cm^2^, so low CO_2_ flow rates guarantee high HER. To operate at a current density of 100 mA/cm^2^ of CO_2_R to CO on a 5 cm^2^ electrode in neutral conditions, at least 7 sccm of CO_2_ must be fed to the reactor.

From a scaleup perspective, the optimal flowrate depends on complex tradeoffs, including between electrolyzer energy and separator capital cost, or selectivity versus crossover^24^. CO_2_R has been shown to be severely limited by CO_2_ availability in a variety of system configurations^10,20,28^. Continuum modeling of current electrolyzer designs suggests that concentration gradients on the cathode surface effectively make it impossible to co-optimize conversion and selectivity at low flow rates^28^. In contrast, large CO_2_ feeds steer selectivity towards CO_2_R but increase separation and compression energies by diluting products and requiring recycle streams. Several reports^19,20,22^ show a parabolic trend in product concentration versus feed flow rate. Further techno-economic analysis is needed to determine whether the process energy and cost are optimal at the peak of this parabola. We also note that industrial CO_2_ feeds differ from most reported experiments. Although our main conclusions are likely transferable, the scale of recycle streams and separation units for a dilute CO_2_ feed could change the optimum between separations and reactions.

Lastly, to clarify reports of high SPC, we recommend reporting FE_CO2R_ and SPC at the same conditions, rather than the best-case scenarios for each. It has been repeatedly shown that selectivity and SPC trade off in current MEA designs^23,29^. A singlestar plot should not include the best performance of an electrolyzer under multiple operating conditions. Given the (bi)carbonate equilibrium, we also note that conversions should always be reported as the ratio of CO_2_ in CO_2_R products/CO_2_ fed, and never CO_2_ consumed/CO_2_ fed.

In summary, we analyzed stream compositions for state-of-the-art CO_2_ electrolyzers to show that single-pass conversion alone does not capture the extent of downstream gas separations required. Although electrolyzers with high SPC do not dilute products in unreacted CO_2_, they still produce mixed cathode product streams. In current electrolyzer designs, this tradeoff arises from physical limitations. Low CO_2_ feed flow rates and proton-rich environments maximize SPC but increase the side reaction of hydrogen evolution. We recommend that researchers prioritize and report outlet gas compositions, since maximizing product yield and mole fraction in the cathode outlet is more practically important than maximizing single-pass conversion. While reactor designs that decouple the tradeoff between selectivity and SPC could be pursued, electrolyzer energy dominates separation for both low and high SPC operation, so this is not the most pressing challenge facing CO_2_R scaleup. Thermocatalytic processes and solid oxide electrolytic cells often operate at low SPC with separation and recycle schemes, optimizing systems for the reaction rather than separation. CO_2_ electrolyzers can similarly benefit from prioritizing other goals, including high product yields and low cell voltages, over increased single-pass conversion.

### Supplementary information

Supplementary Information

### Source data

Source Data

## Data Availability

The source data from this study can be found in the Source Data file (Excel workbook). The workbook includes: literature data, assumptions and calculated metrics used to generate Fig. 2; additional figures comparing metrics; assumptions and analysis for the energy breakdown in Fig. 1e; calculations for limiting CO_2_ flow rates and current densities recommended in the main text. Figure 1a, b are discussed in the Supplementary Information, Section 1, and their source data is available from the authors upon reasonable request. The Supplementary Information details the assumptions made, including in Section 5 and Tables S1, S2. Source data are provided with this paper.
