# Supplementary material for: Maximizing single-pass conversion does not result in practical readiness for CO2 reduction electrolyzers
Source: Nat Commun. 2023 Sep 7;14:5513. doi: 10.1038/s41467-023-41348-w (PMC10484981; doi:10.1038/s41467-023-41348-w)
Supplement: Supplementary file 1 — Supplementary Information [file 41467_2023_41348_MOESM1_ESM.pdf]

Supplementary Information

Maximizing single-pass conversion does not result in practical readiness for CO_2 reduction electrolyzers

Shashwati C. da Cunha[1], Joaquin Resasco[1]*

1. McKetta Department of Chemical Engineering, The University of Texas at Austin, Austin, TX 78712, USA

* Corresponding author: resasco@utexas.edu

Contents

1 Mass balances for sample stream compositions (Figures 1b and 1c) 3

2 Energy requirements for sample electrolyzers (Figure 1e) 6

2.1 Electrolyzer energy . . . 7

2.1.1 Thermodynamics . . . 8

2.1.2 Ohmic loss . . . 8

2.1.3 Overpotential . . . 9

2.2 Separation energy . . . 10

2.2.1 Thermodynamics . . . 10

2.2.2 Inefficiency . . . 11

3 Stream compositions for literature data (Figure 2) 11

3.1 Molar flow rates . . . 11

3.1.1 Product flow rates . . . 11

3.1.2 CO_2 flow rates . . . 12

3.1.3 CO_2 crossover in alkaline electrolytes . . . 13

3.2 Product concentrations . . . 14

3.3 Single-pass conversion 14

4 Additional correlations between CO_2R performance metrics **15**

4.1 Single-pass utilization as a metric 15

4.2 Additional data visualizations 16

5 List of constants **18**

6 List of symbols used **18**

Supplementary References **20**

List of Tables

S1 Assumptions for high FE_{CO2R} and high SPC scenarios in Figure 1 4

S2 Voltage modeling data for overpotential estimation[7] 9

List of Figures

S1 **Energy requirements for a CO_2 electrolyzer vs gas separations.** (a) Total energy requirements, reproduced from Figure 1e. (b) Minimum (thermodynamic) energy loads for reaction vs separation. 7

S2 **SPC and FE_{CO2R} as a function of experimental variables.** (a) High SPC has been achieved in the literature by restricting the feed flow rate, which increases selectivity towards hydrogen evolution. Note that the flow rate axis has been truncated to highlight high SPC datapoints. (Data is also reported in Figure 2b, with axes inverted.) (b) High SPC is not necessarily correlated with high product yield; note that yield is mostly controlled by the current density towards CO_2R. (c) Selectivity towards CO_2R drops at very low inlet flow rates.[2,3,8–34] 16

S3 **Effects of pH on outlet stream compositions.** This figure reproduces Figures 2a, 2c-e with additional information on electrolyte pH. At acidic pH, carbonate crossover is entirely suppressed to yield a pure O_2 stream at the anode outlet, most obvious in (d) here. However, cathode product concentration suffers as hydrogen evolution is strongly promoted by the acidic environment, as (c) shows here. Assumptions made regarding alkaline electrolytes do not fully reflect carbonate crossover at high pH; further details can be found in Section 3.1.3. (a) Product concentration in cathode outlet as a function of SPC (see Figure 2a). (b) Faradaic efficiency to CO_2R products as a function of SPC (see Figure 2c). (c) Product concentration in cathode outlet as a function of Faradaic efficiency. (see Figure 2d). (d) Anode outlet oxygen concentration as a function of SPC (see Figure 2e).[2,3,8–34] 17

1 Mass balances for sample stream compositions (Figures 1b and 1c)

To calculate the cathode and anode gas compositions for the example scenarios in Figures 1b and 1c, the following molar flow rates must be determined:

- CO produced: $\dot{N}_{CO,\, cathode\, outlet} \equiv \dot{N}_{CO}$
- H_2 produced: $\dot{N}_{H_2,\, cathode\, outlet} \equiv \dot{N}_{H_2}$
- O_2 produced: $\dot{N}_{O_2,\, anode\, outlet} \equiv \dot{N}_{O_2}$
- CO_2 fed: $\dot{N}_{CO_2,\, cathode\, inlet}$
- CO_2 crossed over to anode: $\dot{N}_{CO_2,\, anode\, outlet} \equiv \dot{N}_{CO_2,\, crossed}$
- Unreacted residual CO_2 in cathode outlet: $\dot{N}_{CO_2,\, cathode\, outlet} \equiv \dot{N}_{CO_2,\, residual}$

For both cases, the total product molar flow rate ($\dot{N}_{products} \equiv \dot{N}_{CO} + \dot{N}_{H_2}$) is assumed to be 100 sccm. This assumption is equivalent to assuming the same total current in both cases, discussed below. The arbitrary basis is used to illustrate stream composition at a constant total current, as shown by Eqs. (7) - (9). Additional assumptions are listed in Table S1.

Table S1: Assumptions for high FE_{CO2R} and high SPC scenarios in Figure 1

**Parameter**	**High FE_{CO2R}**	**High SPC**
Product flow rate, $\dot{N}_{products}$ (sccm)	100	100
FE_{CO}	99%	60%
SPC	40%	99%
CO_2 crossover, c (mol CO_2 per mol e^-)	0.5	0

The Faradaic efficiency to CO_2R for the high FE_{CO2R} case was chosen based on highly selective reactor performance reported by several groups.[1,2] The selected single-pass conversion has also been demonstrated across varied conditions.[3] Figure 2c in the main text illustrates that these conditions are realistic for a CO_2-to-CO electrolyzer. On the other hand, the high SPC case uses a higher SPC than has been demonstrated thus far, as well as a relatively high FE_{CO} for the given SPC. Such high selectivity is yet to be demonstrated at such high SPC, especially in the acidic cathode conditions needed to suppress crossover to 0%. Therefore, Figure 1 compares the stream composition for a realistic electrolyzer at high FE_{CO2R} to a scenario better than the state-of-the-art for high SPC.

Since CO_2 and H_2 are both 2-e^- products, their Faradaic efficiencies (FE_i) in % are equivalent to their mole fraction of product flow rate:

$$j_{CO} = FE_{CO} \times j = FE_{CO} \times (j_{CO} + j_{H_2}) \tag{1}$$

Substituting Faraday's law,

$$j_i = n_i \times F \times 1000 \times \dot{N}_i \tag{2}$$

$$\implies 2 \times F \times 1000 \times \dot{N}_{CO} = FE_{CO} \times (2 \times F \times 1000 \times \dot{N}_{CO} + 2 \times F \times 1000 \times \dot{N}_{H_2}) \tag{3}$$

$$\implies FE_{CO} = \frac{\dot{N}_{CO}}{\dot{N}_{CO} + \dot{N}_{H_2}} = \frac{\dot{N}_{CO}}{\dot{N}_{products}} = x_{CO} \tag{4}$$

$$\implies \dot{N}_{CO} = FE_{CO} \times \dot{N}_{products} \tag{5}$$

and,

$$\implies \dot{N}_{H_2} = FE_{H_2} \times \dot{N}_{products} \tag{6}$$

These equations also show that the assumption of constant product flow rate equates to constant current

across the high FE_{CO2R} and high SPC scenarios:

Given

$$j_{high\,FE} = j_{high\,SPC} \tag{7}$$

$$\implies (j_{CO} + j_{H_2})_{high\,FE} = (j_{CO} + j_{H_2})_{high\,SPC} \tag{8}$$

dividing by $2 \times F \times 1000$,

$$\implies (\dot{N}_{products})_{high\,FE} = (\dot{N}_{products})_{high\,SPC} \tag{9}$$

The constant current assumption made throughout Figure 1 matches the galvanostatic mode in which electrolyzers are typically tested in lab settings. We note that numeric results will be slightly different for the assumption of the same applied voltage between scenarios. However, our key takeaways remain unchanged. For example, the energy consumed on a per product basis remains higher for the high SPC scenario compared to the high FE_{CO2R} case. This is because the same electrolyzer voltage goes into making a smaller amount of desired products, and electrolyzer energy dominates the total energy load for the process.

The anodic OER reaction rate is assumed to exactly balance the cathodic reduction reaction rate. Since oxygen is a 4-e^- product while H_2 and CO_2 are both 2-e^-, the flow rate of oxygen produced at the anode is $\frac{1}{2}$ the flow rate of cathode products:

$$\dot{N}_{O_2} = \frac{1}{2} \times (\dot{N}_{CO_2} + \dot{N}_{H_2}) = 50\,\text{sccm} \tag{10}$$

The assumed SPC is used to calculate the CO_2 feed to the reactor in a single pass (that is, the fresh feed combined with recycled CO_2):

$$SPC = \frac{\dot{N}_{CO}}{\dot{N}_{CO_2,\,cathode\,inlet}} \tag{11}$$

$$\implies \dot{N}_{CO_2,\,cathode\,inlet} = \frac{\dot{N}_{CO}}{SPC} \tag{12}$$

The assumed crossover, c is used to calculate CO_2 in the anode stream. In the case of high SPC, we assume that no crossover occurs, which can be facilitated by an acidic electrolyte or bipolar membrane. In the high FE_{CO2R} case, we use c = 0.5 mol CO_2 crossover per mol e^- (typical for neutral electrolytes). We

choose to define crossover on a per mol e$^-$ basis rather than as a fraction of the feed, since it reflects the hoomgeneous carbonate reactions. The generation of hydroxide ions arises from the redox reaction rates, and results in the generation of carbonate ions:

$$CO_2 + H_2O + \mathbf{2e}^- \rightarrow CO + 2OH^- \tag{13}$$

$$CO_2 + 2OH^- \rightleftharpoons HCO_3^- + OH^- \rightleftharpoons \mathbf{CO_3^{2-}} + H_2O \tag{14}$$

Therefore, the high $\mathrm{FE_{CO2R}}$ case results in $\frac{1}{2}$ the moles of CO_2 crossed as moles of reaction products formed (see also Eq. 65):

$$\dot{N}_{CO_2,\, crossed} = \frac{\text{mol e}^-}{\text{s}} \times \frac{\text{mol CO}_2\text{ crossed}}{\text{mol e}^-} \tag{15}$$

$$= \frac{j \times A}{F \times 1000} \times c \tag{16}$$

$$= \dot{N}_{products} \times 0.5 = 50\,\text{sccm} \tag{17}$$

The unreacted CO_2 remaining in the cathode outlet stream is calculated by a mass balance on carbon, assuming no accumulation:

$$\dot{N}_{CO_2,\, cathode\, inlet} = \dot{N}_{CO} + \dot{N}_{CO_2,\, residual} + \dot{N}_{CO_2,\, anode\, outlet} \tag{18}$$

$$\implies \dot{N}_{CO_2,\, residual} = \dot{N}_{CO_2,\, cathode\, inlet} - \dot{N}_{CO_2,\, crossed} - \dot{N}_{CO} \tag{19}$$

We do not quantitatively estimate the liquid water streams at the anode inlet and outlet; they are not drawn to scale in Figure 1. Given the wide range in electrolyte flow rates used in literature, they serve only to indicate the excess of liquid water flowing through the anode.

2 Energy requirements for sample electrolyzers (Figure 1e)

The energy analysis here does not account for all energy demands in a theoretical electrolyzer system. For example, voltage drops from pH gradients across the cell and mass transport at both electrodes are neglected. Separations are assumed to take place at 40°C. Despite the simplifications, it is clear that electrolyzer energy dominates separation energy. A comparison of purely thermodynamic minimum energy

requirements for reaction vs separation (Figure S1b), as well as analyses from other groups[4,5] reach the same conclusion. Since electrolyzer energy is an order of magnitude higher than separation energy, the expenditure of electrolyzer energy on making undesirable products (H_2) consumes more energy per unit product, even if separation loads are reduced.

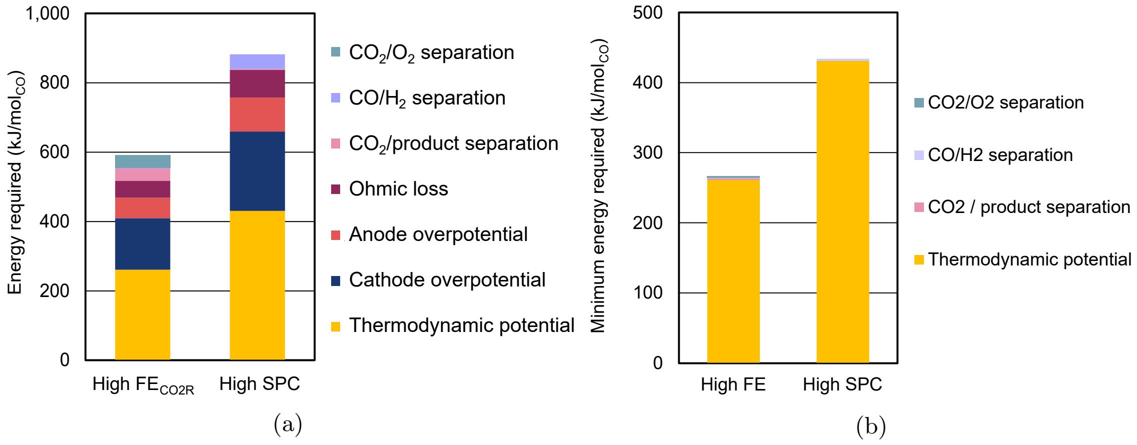

Figure S1: **Energy requirements for a CO_2 electrolyzer vs gas separations.** (a) Total energy requirements, reproduced from Figure 1e. (b) Minimum (thermodynamic) energy loads for reaction vs separation.

Energy loads are calculated for CO_2R to CO per mole of product. A basis of constant CO production rate is assumed; since energy is reported per unit product, this basis is arbitrary. Values for FE_{CO2R}, SPC, and c from Figures 1b and 1c are used for the high FE_{CO2R} and high SPC scenarios (Table S1). Electrolyzer area is calculated to maintain a total current density of 250 mA/cm^2. Given the other assumptions made, increasing the total current density increases ohmic resistance, so our choice of a relatively low j underestimates electrolyzer energy.

2.1 Electrolyzer energy

Electrolyzer energy is estimated from cell potential:

$$E_{cell} = E^0 + \eta_{total} + iR_\Omega \tag{20}$$

where E^0 is the standard cell potential, η_{total} is the combined anode and cathode overpotential, and iR_Ω is the Ohmic drop.

2.1.1 Thermodynamics

Thermodynamic energy requirements are calculated based on standard reduction potentials[6]:

$$E^0_{CO_2R} = -0.11\,\text{V} \tag{21}$$

$$E^0_{OER} = +1.23\,\text{V} \tag{22}$$

$$E^0_{cell} = E^0_{CO_2R} - E^0_{OER} = -1.34\,\text{V} \tag{23}$$

2.1.2 Ohmic loss

Ohmic loss is estimated assuming a membrane electrode assembly configuration. This is a reasonable lower limit on electrolyzer resistance. A 50 μm membrane with a conductivity of 0.5 S m^{-1} is assumed, following the assumptions made by Weng et al.[6]:

$$R_\Omega = \frac{\rho \times L}{A} \tag{24}$$

$$= \frac{\frac{1}{0.5} \times 5 \times 10^{-6}}{\frac{A}{10^4}} \tag{25}$$

where

$$A = \frac{i}{j} = \frac{i}{250\,\text{mA/cm}^2} \tag{26}$$

Total current i is estimated from the CO production rate:

$$i_{CO} = 2 \times F \times 1000 \times \dot{N}_{CO} \tag{27}$$

$$i = \frac{i_{CO}}{FE_{CO}} \tag{28}$$

This gives an Ohmic loss of:

$$E_\Omega = \frac{i}{1000} \times R_\Omega \tag{29}$$

2.1.3 Overpotential

Overpotentials are estimated for the CO_2R and OER half-reactions following the Tafel analysis used by Shin et al.[7]:

$$\eta_{cathode} = TS_{CO_2R} \times \log_{10}(\frac{i_{CO}}{i_{ref,\,CO_2R}}) + \eta_{ref,\,CO_2R} \tag{30}$$

$$\eta_{anode} = TS_{OER} \times \log_{10}(\frac{i_{O_2}}{i_{ref,\,OER}}) + \eta_{ref,\,OER} \tag{31}$$

The following reference values are used, based on the work of Shin et al.[7]:

Table S2: Voltage modeling data for overpotential estimation[7]

**Parameter**	**Value**	**Unit**
Cathodic reference overpotential, $\eta_{ref,\,CO_2R}$	-0.44	V
Anodic reference overpotential, $\eta_{ref,\,OER}$	0.247	V
Cathode Tafel slope, TS_{CO_2R}	-0.23	V/dec
Anode Tafel slope, TS_{OER}	0.041	V/dec
Reference cathode current density, $i_{ref,\,CO_2R}$	10	mA/cm^2
Reference anode current density, $i_{ref,\,OER}$	10	mA/cm^2

Partial currents, i_i, are estimated as follows:

$$i_{O_2} = i \tag{32}$$

$$i_{H_2} = i - i_{CO} \tag{33}$$

Substituting the relevant currents in in Eq. 30 and Eq. 31 gives the cathodic and anodic overpotentials. Estimated cell potentials are in the range of 2.6 V, comparable to other works[4,6,7].

2.2 Separation energy

Outlet flow rates can be estimated as in Section 1:

$$\dot{N}_{O_2} = \frac{i}{4 \times F \times 1000} \tag{34}$$

$$\dot{N}_{H_2} = \frac{i}{2 \times F \times 1000} \tag{35}$$

$$\dot{N}_{CO_2,\, crossed} = \frac{j \times A}{F \times 1000} \times c \tag{36}$$

$$\dot{N}_{CO_2,\, cathode\, inlet} = \frac{\dot{N}_{CO}}{SPC} \tag{37}$$

$$\implies \dot{N}_{CO_2,\, residual} = \dot{N}_{CO_2,\, cathode\, inlet} - \dot{N}_{CO_2,\, crossed} - \dot{N}_{CO} \tag{38}$$

The total stream flow rates for the cathode and anode are calculated as:

$$\dot{N}_{anode\, outlet} = \dot{N}_{O_2} + \dot{N}_{CO_2,\, crossed} \tag{39}$$

$$\dot{N}_{cathode\, outlet} = \dot{N}_{CO_2R\, gas\, products} + \dot{N}_{H_2} + \dot{N}_{CO_2,\, residual} \tag{40}$$

2.2.1 Thermodynamics

The minimum energy for a binary gas separation is calculated for an isothermal and isobaric separation process:

$$W_{min} = \frac{1}{1000} \times R \times T \times \sum_i x_i \times \ln x_i \tag{41}$$

$$= \frac{1}{1000} \times R \times T \times (x_i \times \ln x_i + (1 - x_i) \times \ln(1 - x_i)) \tag{42}$$

where

$$x_i = \frac{\dot{N}_i}{\dot{N}_{stream}} \tag{43}$$

Pairwise separations are considered in the following order:

1. CO_2 from cathode gas, i.e. CO_2 from products
2. CO from H_2

A single separation between CO_2 and O_2 is used at the anode.

2.2.2 Inefficiency

The thermodynamic limit on separation energy is adjusted by an assumed second-law efficiency, following the method used by Moore et al.[5]:

$$W = \frac{W_{min}}{\zeta} \tag{44}$$

Equation 44 gives the work done per mole of stream separated. Energies in Figure 1e are reported per mole of product, i.e. scaled by $\frac{\dot{N}_{stream}}{\dot{N}_{CO}}$.

3 Stream compositions for literature data (Figure 2)

For all references[2,3,8–34] used to generate Figure 2 in the main text, species flow rates are estimated from the reported partial current densities and feed flow rates. The method follows the equations from previous sections, with the added complexity of multiple products, including multicarbon products, for CO_2R over Cu catalysts. Equations are detailed below, extremely similar to those described above.

3.1 Molar flow rates

3.1.1 Product flow rates

$$\dot{N}_{H_2} = \frac{j \times FE_{H_2} \times A}{2 \times F \times 1000} \tag{45}$$

$$\dot{N}_{CO_2R\,products} = \sum_i \frac{j \times FE_i \times A}{n_i \times F \times 1000} \tag{46}$$

$$= \frac{j \times A}{F \times 1000} \times \sum_i \frac{FE_i}{n_i} \tag{47}$$

For CO_2R to CO, this simplifies to:

$$\dot{N}_{CO_2R\,products} = \frac{j \times FE_{CO} \times A}{2 \times F \times 1000} \tag{48}$$

For CO_2R to C_{2+} products, we define a weighted average parameter $\bar{n}_{CO_2R}$ to simplify calculations:

$$\bar{n}_{CO_2R} \equiv \frac{1}{\sum_{i,\, CO_2R} \frac{FE_i}{n_i}} \tag{49}$$

and,

$$\bar{n}_{CO_2R\, gas\, products} \equiv \frac{1}{\sum_{i,\, CO_2R\, gas\, products} \frac{FE_i}{n_i}} \tag{50}$$

$$\implies \dot{N}_{CO_2R} = \frac{j \times A}{\bar{n}_{CO_2R} \times F \times 1000} \tag{51}$$

and,

$$\implies \dot{N}_{CO_2R\, gas\, products} = \frac{j \times A}{\bar{n}_{CO_2R\, gas\, products} \times F \times 1000} \tag{52}$$

At the anode,

$$\dot{N}_{O_2} = \frac{j \times A}{4 \times F \times 1000} \tag{53}$$

3.1.2 CO_2 flow rates

CO_2 flowrates can be estimated using the inlet flow rate, current density to CO_2R products and extent of carbonate crossover.

$$\dot{N}_{CO_2,\, reacted} = \sum_{i,\, CO_2R} \dot{N}_{products} \times \frac{\text{mol CO}_2\text{ consumed}}{\text{mol } i} \tag{54}$$

$$= \sum_{i,\, CO_2R} \frac{j \times A \times z_i}{n_i \times F \times 1000} \tag{55}$$

$$= \frac{j \times A}{F \times 1000} \times \sum_{i,\, CO_2R} \frac{z_i \times FE_i}{n_i} \tag{56}$$

$$\dot{N}_{CO_2,\, crossed} = \frac{\text{mol e}^-}{\text{s}} \times \frac{\text{mol CO}_2\text{ crossed}}{\text{mol e}^-} \tag{57}$$

$$= \frac{j \times A}{F \times 1000} \times c \tag{58}$$

The residual CO_2 in the cathode outlet is calculated by mass balance:

$$\dot{N}_{CO_2,\, residual} = \dot{N}_{CO_2,\, cathode\, inlet} - \dot{N}_{CO_2,\, crossed} - \dot{N}_{CO_2,\, reacted} \tag{59}$$

The cathode inlet flowrate is typically reported in sccm. There are no universally defined standard conditions used to define 1 sccm. Unless otherwise specified, a temperature of 273.15 K and pressure of 1 atm were used. Some works[9,19,24,27,28,32] explicitly cite their standard conditions, while conditions for others[16,23,25,26] were assumed based on reported SPC. We note that in some cases, the value of SPC is quite sensitive to the conversion units. However, all trends discussed in this work hold even if 298.15 K and 1 bar are standard conditions, which can be tested by modifying the Source Data workbook.

$$P \times \dot{V} = \dot{N} \times R \times T \tag{60}$$

$$\implies \dot{V} = \frac{\dot{N} \times R \times T}{P} \tag{61}$$

$$1 \text{ sccm} = 1 \frac{\text{cm}^3}{\text{min}} = 7.44 \times 10^{-7} \frac{\text{mol}}{\text{s}} \tag{62}$$

For some cases, carbonate crossover is reported as a percentage of consumed CO_2, $C\%$. We convert this percentage into a mol CO_2/ mol e$^-$ basis, c:

$$C\% = \frac{\dot{N}_{CO_2,\,crossed}}{\dot{N}_{CO_2,\,crossed} + \dot{N}_{CO_2,\,reacted}} \tag{63}$$

$$= \frac{j \times c}{j \times c + j \times \sum \frac{z_i \times FE_i}{n_i}} \tag{64}$$

$$\implies c = \frac{C\% \times \sum \frac{z_i \times FE_i}{n_i}}{1 - C\%} \tag{65}$$

3.1.3 CO_2 crossover in alkaline electrolytes

Note that we assume that 100% of carbonate ions are reconverted into CO_2 at the anode. This assumption is not strictly true in alkaline electrolytes, especially during startup. Initially, carbonate ions will lower the anolyte pH, transforming hydroxide ions into (bi)carbonate. After steady state is achieved, assuming that the electrolyte is recycled, carbonate will regenerate CO_2 stoichiometrically at the anode as in a neutral electrolyte. This transformation has been experimentally detailed by Ma et al.[21] and Mardle et al[9]. However, if the electrolyte is regenerated to preserve the selectivity achievable in alkaline conditions, CO_2 will never be regenerated in the anode gas, unlike our assumption here.

The nuance of carbonate generation rates vs CO_2 regeneration rates is dependent on the operation mode, and is beyond the scope of our analysis. Instead, we use an intermediate value for the crossover, c, in alkaline solutions (0.65 mol CO_2/ mol e^-), and assume that crossed CO_2 is regenerated. This is likely to underestimate the CO_2 lost from the cathode gas, and overestimate the CO_2 regenerated at the anode. Therefore, both the cathode and anode product concentrations in alkaline electrolytes may be higher than reported here. As we note in the main text, more common reporting of gas stream compositions in a range of operating conditions would be a valuable addition to the field.

3.2 Product concentrations

Having fully determined the gas stream flow rates, we can calculate the concentrations of products in the cathode and anode outlet gases:

$$\dot{N}_{anode\ outlet} = \dot{N}_{O_2} + \dot{N}_{CO_2,\ crossed} \tag{66}$$

$$\dot{N}_{cathode\ outlet} = \dot{N}_{CO_2R\ gas\ products} + \dot{N}_{H_2} + \dot{N}_{CO_2,\ residual} \tag{67}$$

$$x_{products,\ cathode\ outlet} = \frac{\dot{N}_{products}}{\dot{N}_{cathode\ outlet}} \tag{68}$$

$$x_{O_2,\ anode\ outlet} = \frac{\dot{N}_{O_2}}{\dot{N}_{anode\ outlet}} \tag{69}$$

$$\tag{70}$$

3.3 Single-pass conversion

When SPC is not reported explicitly, the feed flow rate and current density to all CO_2R products are combined to calculate SPC:

$$SPC = \frac{\dot{N}_{CO_2R}}{\dot{N}_{CO_2,\ cathode\ inlet}} \tag{71}$$

4 Additional correlations between CO_2R performance metrics

4.1 Single-pass utilization as a metric

Single-pass utilization (SPU) is an alternative representation of the relative consumption of reactant by CO_2R vs homogeneous reactions:

$$SPU = \frac{\dot{N}_{CO_2,\,reacted}}{\dot{N}_{CO_2,\,crossed} + \dot{N}_{CO_2,\,reacted}} \tag{72}$$

$$\implies SPU = 1 - C\% \tag{73}$$

Although SPU indicates the extent of reactant loss towards carbonate reaction, it suffers from similar limitations as SPC. For example, Figures 1b and 1c contrast scenarios with low vs high SPC. These two situations also correspond to low vs high SPU:

- Figure 1b: 90% FE_{CO2R}, 40% SPC, 50% CO_2 crossover = **50% SPU**. The resulting product stream is **67% CO**.
- Figure 1c: 60% FE_{CO2R}, 99% SPC, 0% CO_2 crossover = **100% SPU**. The resulting product stream is only **60% CO**.

Since SPU does not account for selectivity losses to HER, it does not reflect the product composition in the cathode outlet. While the extent of CO_2 contaminating the anode gas is conveyed by SPU, that information is more intuitively presented as the anode gas composition. Stream composition is also a common measurement across chemical processes that can be assessed without a comprehensive understanding of the CO_2R reaction mechanism, unlike SPU.

Another illustrative example where low SPU poses no practical issues is in the case of a liquid oxidation reaction at the anode. If the anode product is exclusively in the liquid phase, a low SPU results in a pure CO_2 stream at the anode outlet, which can be recycled directly. If a bipolar membrane is used to increase SPU in such a system, overall performance suffers as the cell voltage and HER current increase, which raises the opex and lowers product yield.

4.2 Additional data visualizations

In addition to the visualizations presented in Figure 2, we provide additional representations of the data in the following figures. Additional plots as well as raw data for Figure 2, Figure S2, and Figure S3 can be found in the Source Data.

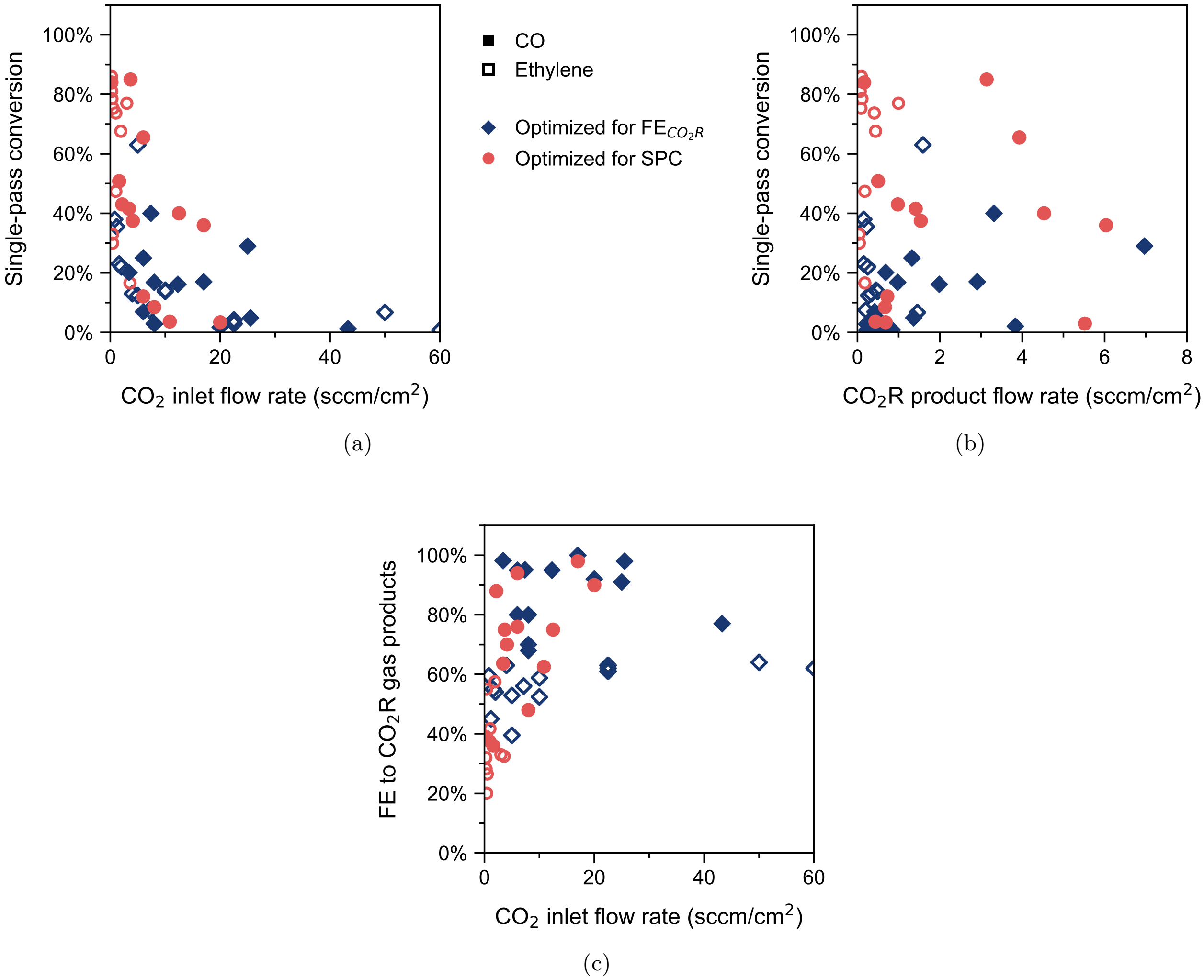

Figure S2: **SPC and $\mathbf{FE_{CO2R}}$ as a function of experimental variables.** (a) High SPC has been achieved in the literature by restricting the feed flow rate, which increases selectivity towards hydrogen evolution. Note that the flow rate axis has been truncated to highlight high SPC datapoints. (Data is also reported in Figure 2b, with axes inverted.) (b) High SPC is not necessarily correlated with high product yield; note that yield is mostly controlled by the current density towards CO_2R. (c) Selectivity towards CO_2R drops at very low inlet flow rates.[2,3,8–34]

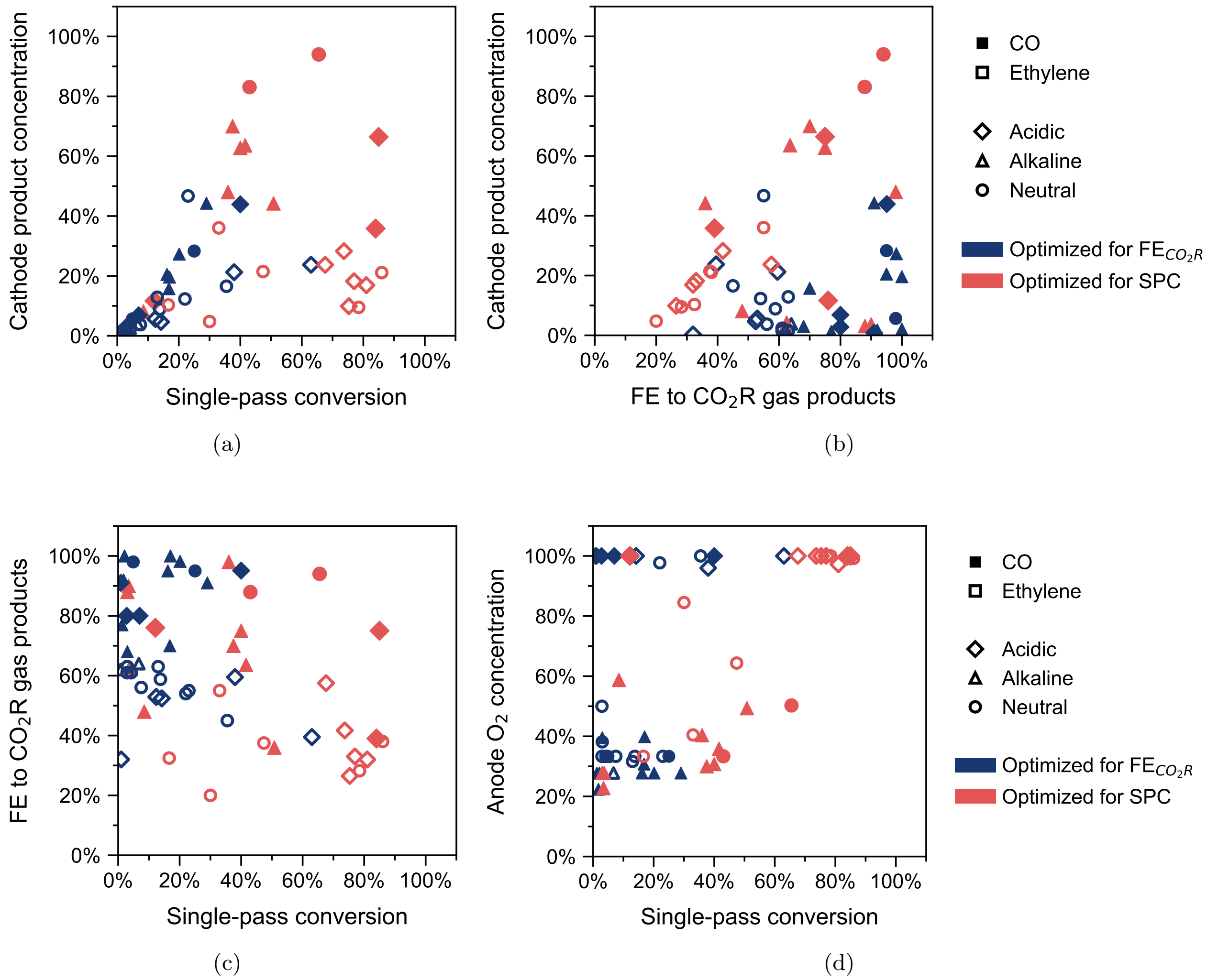

Figure S3: **Effects of pH on outlet stream compositions.** This figure reproduces Figures 2a, 2c-e with additional information on electrolyte pH. At acidic pH, carbonate crossover is entirely suppressed to yield a pure O_2 stream at the anode outlet, most obvious in (d) here. However, cathode product concentration suffers as hydrogen evolution is strongly promoted by the acidic environment, as (c) shows here. Assumptions made regarding alkaline electrolytes do not fully reflect carbonate crossover at high pH; further details can be found in Section 3.1.3.
(a) Product concentration in cathode outlet as a function of SPC (see Figure 2a). (b) Faradaic efficiency to CO_2R products as a function of SPC (see Figure 2c). (c) Product concentration in cathode outlet as a function of Faradaic efficiency. (see Figure 2d). (d) Anode outlet oxygen concentration as a function of SPC (see Figure 2e).[2,3,8–34]

5 List of constants

R	Ideal gas constant	8.314472	J/mol.K
F	Faraday's constant	96485.33	C/mol e^-
T	Temperature at standard state	273.15 or 298.15	K
P	Pressure at standard state	101325	Pa
ζ	Second-law efficiency	7%	

6 List of symbols used

$\dot{N}_{i,j}$	Molar flow rate of species i in stream j	mol/s
$\dot{V}_i$	Volumetric flow rate	m^3/s
i	Total current	mA
A	Cathode area	cm^2
j	Total current density	mA/cm^2
j_i	Partial current density to species i	mA/cm^2
FE_i	Faradaic efficiency to species i	
SPC	Single-pass conversion of CO_2 into CO_2R products	
n_i	Electrons transferred per mole of species i	mol e^-/mol i
z_i	Moles of CO_2 consumed per mole of species i (stoichiometric carbon ratio)	mol CO_2/mol i

c	Crossover ratio, moles of CO_2 crossed to anode per mole of electrons transferred	mol CO_2/mol e^-
$C\%$	Fraction of consumed CO_2 crossed to anode	
E_i	Potential drop involved in i	V
E^0	Standard reduction potential	V
W	Work done	kJ/mol
x_i	Mole fraction of species i	
R_Ω	Ohmic resistance	Ω
ρ	Resistivity	1/S·cm
L	Membrane thickness	m

Supplementary References

1. Bondue, C. J., Graf, M., Goyal, A. & Koper, M. T. M. Suppression of Hydrogen Evolution in Acidic Electrolytes by Electrochemical CO $_2$ Reduction. en. *Journal of the American Chemical Society* **143,** 279–285. ISSN: 0002-7863, 1520-5126. https://pubs.acs.org/doi/10.1021/jacs.0c10397 (2022) (Jan. 2021).

2. Liu, Z., Yang, H., Kutz, R. & Masel, R. I. CO_2 Electrolysis to CO and O_2 at High Selectivity, Stability and Efficiency Using Sustainion Membranes. en. *Journal of The Electrochemical Society* **165,** J3371–J3377. ISSN: 0013-4651, 1945-7111. https://iopscience.iop.org/article/10.1149/2.0501815jes (2022) (2018).

3. Jeng, E. & Jiao, F. Investigation of CO_2 single-pass conversion in a flow electrolyzer. en. *Reaction Chemistry & Engineering* **5,** 1768–1775. ISSN: 2058-9883. https://pubs.rsc.org/en/content/articlelanding/2020/re/d0re00261e (2022) (Aug. 2020).

4. Jouny, M., Luc, W. & Jiao, F. General Techno-Economic Analysis of CO_2 Electrolysis Systems. en. *Industrial & Engineering Chemistry Research* **57,** 2165–2177. ISSN: 0888-5885, 1520-5045. https://pubs.acs.org/doi/10.1021/acs.iecr.7b03514 (2023) (Feb. 2018).

5. Moore, T. *et al.* Electrolyzer energy dominates separation costs in state-of-the-art CO_2 electrolyzers: Implications for single-pass CO_2 utilization. en. *Joule* **7,** 782–796. ISSN: 25424351. https://linkinghub.elsevier.com/retrieve/pii/S2542435123001277 (2023) (Apr. 2023).

6. Weng, L.-C., Bell, A. T. & Weber, A. Z. Towards membrane-electrode assembly systems for CO2 reduction: a modeling study. en. *Energy & Environmental Science* **12,** 1950–1968. ISSN: 1754-5692, 1754-5706. http://xlink.rsc.org/?DOI=C9EE00909D (2021) (2019).

7. Shin, H., Hansen, K. U. & Jiao, F. Techno-economic assessment of low-temperature carbon dioxide electrolysis. en. *Nature Sustainability* **4,** 911–919. ISSN: 2398-9629. https://www.nature.com/articles/s41893-021-00739-x (2023) (July 2021).

8. Bhargava, S. S. *et al.* System Design Rules for Intensifying the Electrochemical Reduction of CO_2 to CO on Ag Nanoparticles. en. *ChemElectroChem* **7,** 2001–2011. ISSN: 2196-0216, 2196-0216. https://onlinelibrary.wiley.com/doi/10.1002/celc.202000089 (2022) (May 2020).

9. Mardle, P., Cassegrain, S., Habibzadeh, F., Shi, Z. & Holdcroft, S. Carbonate Ion Crossover in Zero-Gap, KOH Anolyte CO_2 Electrolysis. en. *The Journal of Physical Chemistry C* **125,** 25446–25454. ISSN: 1932-7447, 1932-7455. https://pubs.acs.org/doi/10.1021/acs.jpcc.1c08430 (2023) (Nov. 2021).

10. Pan, B. *et al.* Close to 90% Single-Pass Conversion Efficiency for CO_2 Electroreduction in an Acid-Fed Membrane Electrode Assembly. en. *ACS Energy Letters,* 4224–4231. ISSN: 2380-8195, 2380-8195. https://pubs.acs.org/doi/10.1021/acsenergylett.2c02292 (2022) (Oct. 2022).

11. Subramanian, S., Middelkoop, J. & Burdyny, T. Spatial reactant distribution in CO_2 electrolysis: balancing CO_2 utilization and faradaic efficiency. en. *Sustainable Energy & Fuels* **5,** 6040–6048. ISSN: 2398-4902. http://xlink.rsc.org/?DOI=D1SE01534F (2023) (2021).

12. Verma, S. *et al.* Insights into the Low Overpotential Electroreduction of CO_2 to CO on a Supported Gold Catalyst in an Alkaline Flow Electrolyzer. en. *ACS Energy Letters* **3,** 193–198. ISSN: 2380-8195, 2380-8195. https://pubs.acs.org/doi/10.1021/acsenergylett.7b01096 (2022) (Jan. 2018).

13. Wheeler, D. G. *et al.* Quantification of water transport in a CO_2 electrolyzer. en. *Energy & Environmental Science* **13,** 5126–5134. ISSN: 1754-5692, 1754-5706. http://xlink.rsc.org/?DOI=D0EE02219E (2023) (2020).

14. Yang, K. *et al.* Cation-Driven Increases of CO_2 Utilization in a Bipolar Membrane Electrode Assembly for CO_2 Electrolysis. en. *ACS Energy Letters* **6,** 4291–4298. ISSN: 2380-8195, 2380-8195. https://pubs.acs.org/doi/10.1021/acsenergylett.1c02058 (2022) (Dec. 2021).

15. Corral, D. *et al.* Advanced manufacturing for electrosynthesis of fuels and chemicals from CO_2. en. *Energy & Environmental Science* **14,** 3064–3074. ISSN: 1754-5692, 1754-5706. http://xlink.rsc.org/?DOI=D0EE03679J (2023) (2021).

16. Gabardo, C. M. *et al.* Continuous Carbon Dioxide Electroreduction to Concentrated Multi-carbon Products Using a Membrane Electrode Assembly. en. *Joule* **3,** 2777–2791. ISSN: 25424351. https://linkinghub.elsevier.com/retrieve/pii/S2542435119303654 (2022) (Nov. 2019).

17. García de Arquer, F. P. *et al.* CO_2 electrolysis to multicarbon products at activities greater than 1 A cm2. en. *Science* **367,** 661–666. ISSN: 0036-8075, 1095-9203. https://www.science.org/doi/10.1126/science.aay4217 (2022) (Feb. 2020).

18. Edwards, J. P. *et al.* Efficient electrocatalytic conversion of carbon dioxide in a low-resistance pressurized alkaline electrolyzer. en. *Applied Energy* **261,** 114305. ISSN: 03062619. https://linkinghub.elsevier.com/retrieve/pii/S0306261919319920 (2023) (Mar. 2020).

19. Huang, J. E. *et al.* CO_2 electrolysis to multicarbon products in strong acid. en. *Science* **372,** 1074–1078. ISSN: 0036-8075, 1095-9203. https://www.science.org/doi/10.1126/science.abg6582 (2022) (June 2021).

20. Khan, M. *et al.* Zero-crossover electrochemical CO_2 reduction to ethylene with co-production of valuable chemicals. en. *Chem Catalysis* **2,** 2077–2095. ISSN: 26671093. https://linkinghub.elsevier.com/retrieve/pii/S2667109322003360 (2023) (Aug. 2022).

21. Ma, M. *et al.* Insights into the carbon balance for CO_2 electroreduction on Cu using gas diffusion electrode reactor designs. en. *Energy & Environmental Science* **13,** 977–985. ISSN: 1754-5692, 1754-5706. http://xlink.rsc.org/?DOI=D0EE00047G (2021) (2020).

22. Ma, M., Kim, S., Chorkendorff, I. & Seger, B. Role of ion-selective membranes in the carbon balance for CO $_2$ electroreduction *via* gas diffusion electrode reactor designs. en. *Chemical Science* **11,** 8854–8861. ISSN: 2041-6520, 2041-6539. http://xlink.rsc.org/?DOI=D0SC03047C (2022) (2020).

23. O'Brien, C. P. *et al.* Single Pass CO_2 Conversion Exceeding 85% in the Electrosynthesis of Multicarbon Products via Local CO_2 Regeneration. en. *ACS Energy Letters* **6,** 2952–2959. ISSN: 2380-8195, 2380-8195. https://pubs.acs.org/doi/10.1021/acsenergylett.1c01122 (2022) (Aug. 2021).

24. Ozden, A. *et al.* Energy- and carbon-efficient CO_2/CO electrolysis to multicarbon products via asymmetric ion migration–adsorption. en. *Nature Energy.* ISSN: 2058-7546. https://www.nature.com/articles/s41560-022-01188-2 (2023) (Jan. 2023).

25. Xie, K. *et al.* Bipolar membrane electrolyzers enable high single-pass CO_2 electroreduction to multicarbon products. en. *Nature Communications* **13,** 3609. ISSN: 2041-1723. https://www.nature.com/articles/s41467-022-31295-3 (2023) (June 2022).

26. Xie, K. *et al.* Eliminating the need for anodic gas separation in CO2 electroreduction systems via liquid-to-liquid anodic upgrading. en. *Nature Communications* **13,** 3070. ISSN: 2041-1723. https://www.nature.com/articles/s41467-022-30677-x (2022) (Dec. 2022).

27. Xu, Y. *et al.* A microchanneled solid electrolyte for carbon-efficient CO_2 electrolysis. en. *Joule* **6,** 1333–1343. ISSN: 25424351. https://linkinghub.elsevier.com/retrieve/pii/S2542435122001908 (2022) (June 2022).

28. Zhao, Y. *et al.* Conversion of CO_2 to multicarbon products in strong acid by controlling the catalyst microenvironment. en. *Nature Synthesis.* ISSN: 2731-0582. https://www.nature.com/articles/s44160-022-00234-x (2023) (Feb. 2023).

29. Endrődi, B. *et al.* High carbonate ion conductance of a robust PiperION membrane allows industrial current density and conversion in a zero-gap carbon dioxide electrolyzer cell. en. *Energy & Environmental Science* **13,** 4098–4105. ISSN: 1754-5692, 1754-5706. http://xlink.rsc.org/?DOI=D0EE02589E (2022) (2020).

30. Endrődi, B. *et al.* Multilayer Electrolyzer Stack Converts Carbon Dioxide to Gas Products at High Pressure with High Efficiency. en. *ACS Energy Letters* **4,** 1770–1777. ISSN: 2380-8195, 2380-8195. https://pubs.acs.org/doi/10.1021/acsenergylett.9b01142 (2022) (July 2019).

31. Gu, J. *et al.* Modulating electric field distribution by alkali cations for CO_2 electroreduction in strongly acidic medium. en. *Nature Catalysis.* ISSN: 2520-1158. https://www.nature.com/articles/s41929-022-00761-y (2022) (Apr. 2022).

32. Larrazábal, G. O. *et al.* Analysis of Mass Flows and Membrane Cross-over in CO_2 Reduction at High Current Densities in an MEA-Type Electrolyzer. en. *ACS Applied Materials & Interfaces* **11,** 41281–41288. ISSN: 1944-8244, 1944-8252. https://pubs.acs.org/doi/10.1021/acsami.9b13081 (2022) (Nov. 2019).

33. Li, H. *et al.* Tailoring acidic microenvironments for carbon-efficient CO_2 electrolysis over a Ni–N–C catalyst in a membrane electrode assembly electrolyzer. en. *Energy & Environmental Science,* 10.1039.D2EE03482D. ISSN: 1754-5692, 1754-5706. http://xlink.rsc.org/?DOI=D2EE03482D (2023) (2023).

34. Liu, Z. *et al.* Acidic Electrocatalytic CO_2 Reduction Using Space-Confined Nanoreactors. en. *ACS Applied Materials & Interfaces* **14,** 7900–7908. ISSN: 1944-8244, 1944-8252. https://pubs.acs.org/doi/10.1021/acsami.1c21242 (2023) (Feb. 2022).
